# Nanocomposite coating of albumin/Li-containing bioactive glass nanospheres promotes osteogenic activity of PEEK

**DOI:** 10.1007/s10856-021-06597-5

**Published:** 2021-09-08

**Authors:** Xubin Qiu, Ming Zhuang, Xiaofeng Yuan, Zhiwei Liu, Wenjian Wu

**Affiliations:** 1 Department of Orthopaedics, The Third Affiliated Hospital of Soochow University, Changzhou, 213000 Jiangsu China; 2 Department of Orthopedics, Ruijin Hospital, Shanghai Jiaotong University School of Medicine, Shanghai, 200025 China

## Abstract

Polyetheretherketone (PEEK) is an important material applied in orthopedic applications, as it posses favorable properties for orthopedic implants, e.g., radiolucency and suitable elastic modulus. However, PEEK exhibits insufficient osteogenesis and osteointegration that limits its clinical applications. In this study, we aimed to enhance the osteogenisis of PEEK by using a surface coating approach. Nanocomposite coating composed of albumin/lithium containing bioactive glass nanospheres was fabricated on PEEK through dip-coating method. The presence of nanocomposite coating on PEEK was confirmed by SEM, FTIR, and XRD techniques. Nanocomposite coatings significantly enhanced hydrophilicity and roughness of PEEK. The nanocomposite coatings also enhanced adhesion, proliferation, and osteogenic differentiation of bone mesenchymal stem cells due to the presence of bioactive glass nanospheres and the BSA substrate film. The results indicate the great potential of the nanocomposite coating in enhancing osteogenesis and osteointegration of PEEK implants.

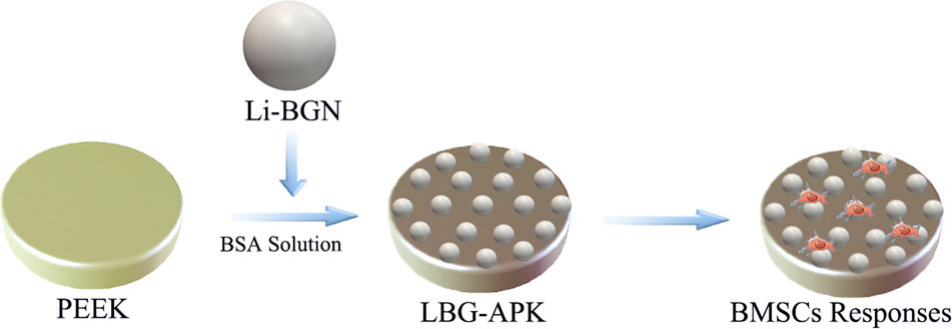

## Introduction

Polyetheretherketone (PEEK) has been widely used as a promising artificial implant material due to its biocompatibility, radiolucency, and similar mechanical properties to bone tissue [1]. However, its low degree of osteointegration caused by the hydrophobic and bioinert surface limits the clinical applications of PEEK in hard tissue replacement area [2]. Various surface modification methods (e.g., spin coating) have been applied to improve the hydrophilicity and bioactivity of PEEK [3]. Bioactive glass nanospheres (BGN) have exhibited great potential as coating on orthopedic implants or scaffolds to enhance their osteogenesis and osteointegration, due to their bone bonding ability, osteogenic, and angiogenic activities [4]. Natural polymers such as chitosan are usually used as a matrix for embedding bioactive particles to form composite coating on PEEK to improve hydrophilicity and bioactivity [5].

Albumin, an endogenous, non-glycosylated protein, is produced predominantly in the liver by hepatocytes and secreted into the blood as a major constituent of plasma. Albumin hydrogel could be formed through pH-induced, thermal induced, salt-induced methods [6]. Albumin hydrogel can act as coating of implants. For example, Sarah et al. prepared coating of bovine serum albumin (BSA) on titanium based implant through electrophoretic deposition [7]. Their results showed that BSA layer could act as an intermediary in interaction with cells, thus leading to enhanced cell adhesion and proliferization. It is thus expected that BGN/BSA composite coating on PEEK can combine the advantages of both materials and promote osteogensis and osteointegration.

It is known that ionic dissolution products of bioactive glasses (BGs) can construct a suitable ion-related microenvironment favorable for the osteogenesis of stem cells [8]. Incorporation of biologically active ions in BGs can improve the biological activities of BGs toward enhanced bone regeneration. It was demonstrated that lithium (Li) ions could promote osteogenesis by activating canonical Wnt/β-catenin pathway [9]. Silicon (Si) is one of the bioactive trace elements in the human body, which localizes in the active calcification sites in young bone and associates with calcium (Ca) in an early stage of calcification [8]. Therefore, degradable Li-containing BG may induce a microenvironment enriched with Si, Ca, and Li ions to improve osteogenesis of cells.

In this study, nanocomposite coatings composed of albumin and Li-containing bioactive glass nanospheres (Li-BGN) were fabricated on PEEK to enhance osteogenic activity. The physical and chemical properties of surface modified PEEK were characterized. The effects of nanocomposite coatings on osteogenisis of mesenchymal stem was evaluated.

## Materials and methods

### Preparation of coating on PEEK samples

PEEK samples (Φ10mm) were purchased from Changzhou Junhua High Performance Polymers Co. LTD. Lithium containing bioactive glass nanospheres, 10% Li-BGN (80SiO_2_-10CaO-10Li_2_O, mol%) was synthesized using a modified Stöber method [10]. Briefly, 18 ml TEOS was added into 72 ml ethanol (96%); then 27 ml ammonia (28%), 48 ml ethanol and water were added. After reaction for 30 min under fast stirring, specific amounts of Ca (NO_3_)_2_·4H_2_O and LiCl were added. The formed colloids were then collected by centrifugation at 7197 rcf for 15 min, and washed twice with deionized water and once with ethanol (96%, VWR). After drying at 60 °C overnight, the samples were calcined at 700 °C for 2 h to obtain Li-BGNs.

To prepare the composition coating on PEEK, Li-BGN was first homogeneously dispersed in BSA solution (10% wt) with MgCl_2_ (1.5%) under ultrasonication. The pH was then adjusted to 4.5 with acetic acid. Then PEEK samples were dipped into the dispersion for 10 s. After drying at 60 °C overnight, the modified PEEK was obtained. The coating of pure BSA (APEEK), 0.5mg/mL Li-BGN containing BSA (5LBG-APK) and 1mg/mL Li-BGN containing BSA (10LBG-APK) were prepared. All chemicals were purchased from Shanghai Macklin Biochemical Co., Ltd.

### Characterization of samples

The structure of samples (PEEK, APEEK, 5LBG-APK, and 10LBG-APK) was characterized by Fourier transform infrared spectrometry (Nicolet 6700, Nicolet, USA), X-ray diffraction (XRD, 18KW/D/max2550VB/PC, Rigaku Co., Japan). The surface morphology of samples was detected by scanning electron microscopy (Hitachi, S4800, Japan). The wettability of the samples were determined using a contact angle meter (JC2000C1, Power each, China).

### Ion release of samples

The changes of ions concentration in solution were evaluated by immersing the samples (APEEK, 5LBG-APK, and 10LBG-APK) into Tris-HCl buffer (10 ml) for 1, 3, and 7 days. The concentrations of Li, Ca, Mg, Si ions in the solution at each time point were determined by Inductivity Coupled Plasma (ICP-OES, Agilent IC, USA).

### Cell responses to samples

#### Cells culture

Rat bone mesenchymal stem cells (BMSCs) were harvested from the tibia and femur of adult Sprague Dawley rats (4–6 weeks) and cultured in α-Modified Eagle’s medium (α-MEM, Hyclone, USA) with the supplement of 10% fetal bovine serum (Thermo Fisher Scientific, USA) at 37 °C under an atmosphere of 95% air and 5% CO_2_, and the medium was changed every 3 days. The samples (PEEK, APEEK, 5LBG-APK, and 10LBG-APK) with the size of φ10 × 2 mm^3^ sterilized by ethylene oxide were used for the cell culture.

#### Cell morphology

The samples were cultured with BMSCs with a density of 2 × 10^4^ cells per well for 24 h, then the samples were fixed with glutaraldehyde for 6 h and rinsed lightly with PBS for twice, stained with Fluorescein Isothiocyanate-Phalloidin (FITC-phalloidin, Roche, USA) for 30 min and 4′,6-diamidino-2-phenylindole (Roche, USA) for 8 min. The morphology of cells was observed by laser scanning confocal microscopy (LSM-800, Zeiss, Germany).

#### Cell adhesion

The adhesion of BMSCs on samples was evaluated by Cell Counting Kit-8 assay (CCK-8, Dojindo, Japan). The samples were placed in 24-well plates. After cultured with BMSCs with a density of 2 × 10^4^ cells per well for 2, 6, and 12 h, the samples were rinsed with PBS for three times to remove the unattached cells and transferred to new 24-well plates. PEEK cultured with BMSCs was used as a control. Then, the working solution composed of CCK-8 solution (40 μl) and α-MEM (400 μl) was added into the well plates and incubated with samples at 37 °C for 2 h. After that, the supernatant was taken to a 96-well plate, and the optical density (OD) was measured at the wavelength of 450 nm using a microplate reader.

#### Cell proliferation

The proliferation of BMSCs was determined by CCK-8 assay. After cultured with BMSCs with a density of 2 × 10^4^ cells per well for 1, 3, and 5 days, the samples were rinsed by PBS solution for twice and transferred to another 24-well plate. The samples were incubated in working solution at 37 °C for 2 h. The supernatant was taken to a 96-well plate, and the OD values were measured at the wavelength of 450 nm using a microplate reader.

#### Expression of osteogenic-related genes

To investigate the osteogenic differentiation of cells on the samples, the expression of four osteogenic-related genes including alkaline phosphatase (ALP), bone sialoprotein (BSP), osteocalcin (OCN), and runt-related transcription factor (Runx2) were examined. BMSCs were seeded on the samples and cultured in 24-well plates at a density of 2 × 10^4^ cells/well for 7, 14, and 21 days. Then total RNA was extracted from the BMSCs by Trizol reagent (Invitrogen, Australia) and chloroform. A reverse transcription procedure was carried out using a High Capacity RNA-to-cDNA kit (Takara Bio, Japan). The expressions of these genes were quantified applying Real-time PCR (ABI Prism 7900HT sequence detection system) with SYBR Green PCR Master Mix (Applied Biosystems, USA). The sequences of primers were shown in Table 1. The expression of the genes was normalized relative to the expression of GAPDH.

**Table 1:** Primers used for RT-PCR analysis

Gene	Forward primer sequence (5′-3′)	Reverse primer sequence (5′-3′)
GAPDH	TGTTCCTACCCCCAATGTATCCG	TGCTTCACCACCTTCTTGATGTCAT
ALP	ACCATTCCCACGTCTTCACATTT	AGACATTCTCTCGTTCACCGCC
BSP	ATGGCCTGTGCTTTCTCAATG	AGGATAAAAGTGGCATGCTTG
Runx2	ACTTCCTGTGCTCGGTGCT	GACGGTTATGGTCAAGGTGAA
OCN	CCTCACACTCCTCGCCCTATT	CCCTCCTGCTTGGACACAAA

### Statistical analysis

Statistical analyses of the data were performed by using the SPSS 15.0 software (SPSS, USA) and expressed as the mean ± standard deviation (*M* ± SD). A minimum of three samples per group was tested for characterization. *p* < 0.05 was considered statistically significant.

## Results

**Fig. 1:**
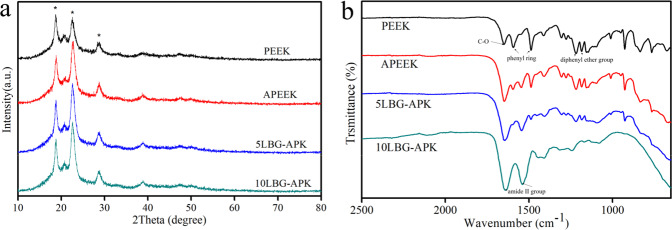
XRD patterns (**a**) and FTIR spectra (**b**) of PEEK, APEEK, 5LBG-APK, and 10LBG-APK

The XRD patterns (a) and FTIR spectra (b) were shown in Fig. 1. Diffraction peaks assigned to (110), (111), (200), and (211) reflections of PEEK were detected among PEEK, APEEK, 5LBG-APK, and 10LBG-APK. No significant difference could be found among these samples. The FTIR results of PEEK, APEEK, 5LBG-APK, and 10LBG-APEEK are shown in Fig. 1b. The intensity of all the absorption bands pertaining to pure PEEK substrate appears much less in the case of the coated specimens. Moreover, the significant absorption bands at around 900 cm^−1^ related to the Si–O–Si can be identified.

**Fig. 2:**
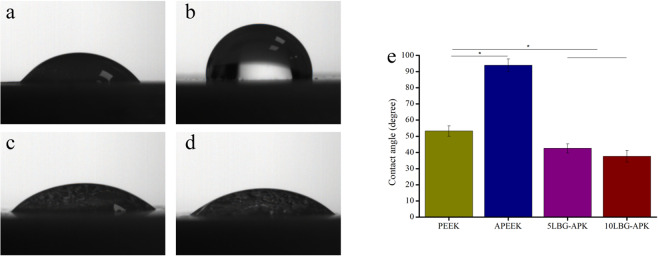
Photos of hydrophilia test of PEEK (a), APEEK (b), 5LBG-APK (c), and 10LBG-APK (d); Quantification of water contact angle for samples (**e**)

The hydrophilia property test of PEEK and modified samples were showed in Fig. 2. The average value of water contact angle of PEEK, APEEK, 5LBG-APK, and 10LBG-APK were 53.2°, 93.9°, 42.5, 37.6° (Fig. 2e), respectively. The coating of pure BSA on PEEK led to a more hydrophobic surface than that of pure PEEK (Fig. 2a–d). The incorporation of LBG in BSA led to a more hydrophilic surface than that of pure PEEK.

**Fig. 3:**
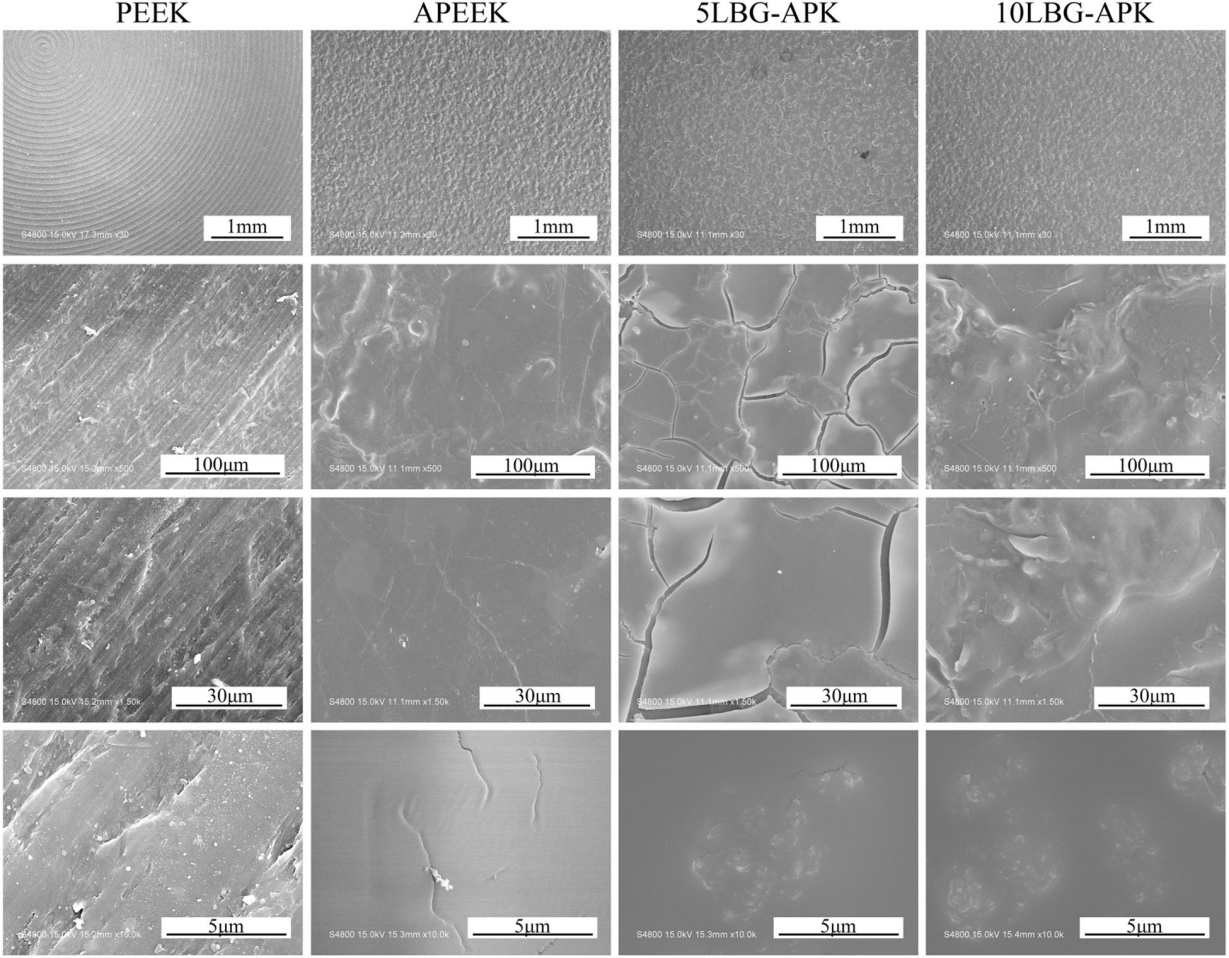
Morphology observation of PEEK, APEEK, 5LBG-APK, and 10LBG-APK by FE-SEM

The morphology of PEEK and modified samples were presented in Fig. 3. After dip-coating, a surface of BSA film was coated on the surface of PEEK. Similar structure was observed on the surface of APEEK, 5LBG-APK, and 10LBG-APK on mm-scale. Massive BSA could be found on the surface of APEEK, 5LBG-APK, and 10LBG-APK on μm-scale. Some LBGN could be found on the surface of 5LBG-APK and 10LBG-APK on nano-scale. The Li-BGN showed homo-diameter and homo-dispersed on the surface of BSA film on PEEK substrate. 10LBG-APK showed much more nanospheres on/in the coating film of BSA.

**Fig. 4:**
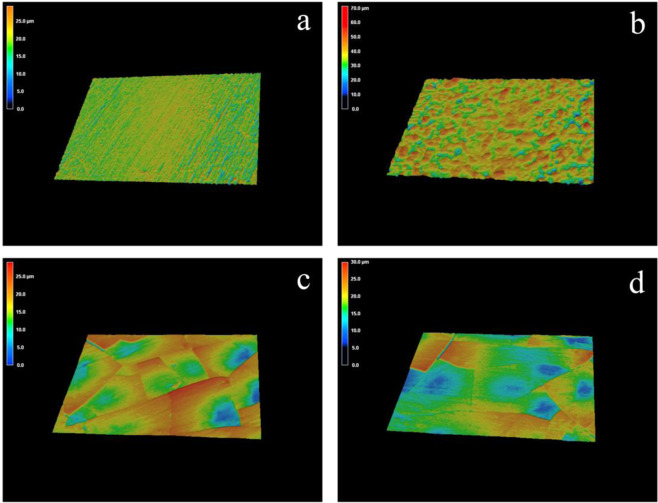
Roughness observation and test of PEEK (**a**), APEEK (**b**), 5LBG-APK (**c**), and 10LBG-APK (**d**) by 3D confocal laser microscopy

The roughness of PEEK, APEEK, 5LBG-APK, and 10LBG-APK were detected by 3D confocal microscopy. The Ra and Rq of different samples were shown in Table 2. After coating with BSA film, the surface of APEEK, 5LBG-APK, and 10LBG-APK were rougher than that of PEEK (Fig. 4). After incorporation of BGN, 5LBG-APK, and 10LBG-APK showed a smoother surface than that of APEEK. No significant difference could be observed in 5LBG-APK and 10LBG-APK.

**Table 2:** Roughness of PEEK, APEEK, 5LBG-APK, and 10LBG-APK

Roughness	PEEK	APEEK	5LBG-APK	10LBG-APK
Ra (μm)	1.9 ± 0.2	4.1 ± 0.3	3.2 ± 0.2	3.0 ± 0.4
Rq (μm)	2.4 ± 0.3	5.2 ± 0.1	4.2 ± 0.1	3.7 ± 0.3

**Fig. 5:**
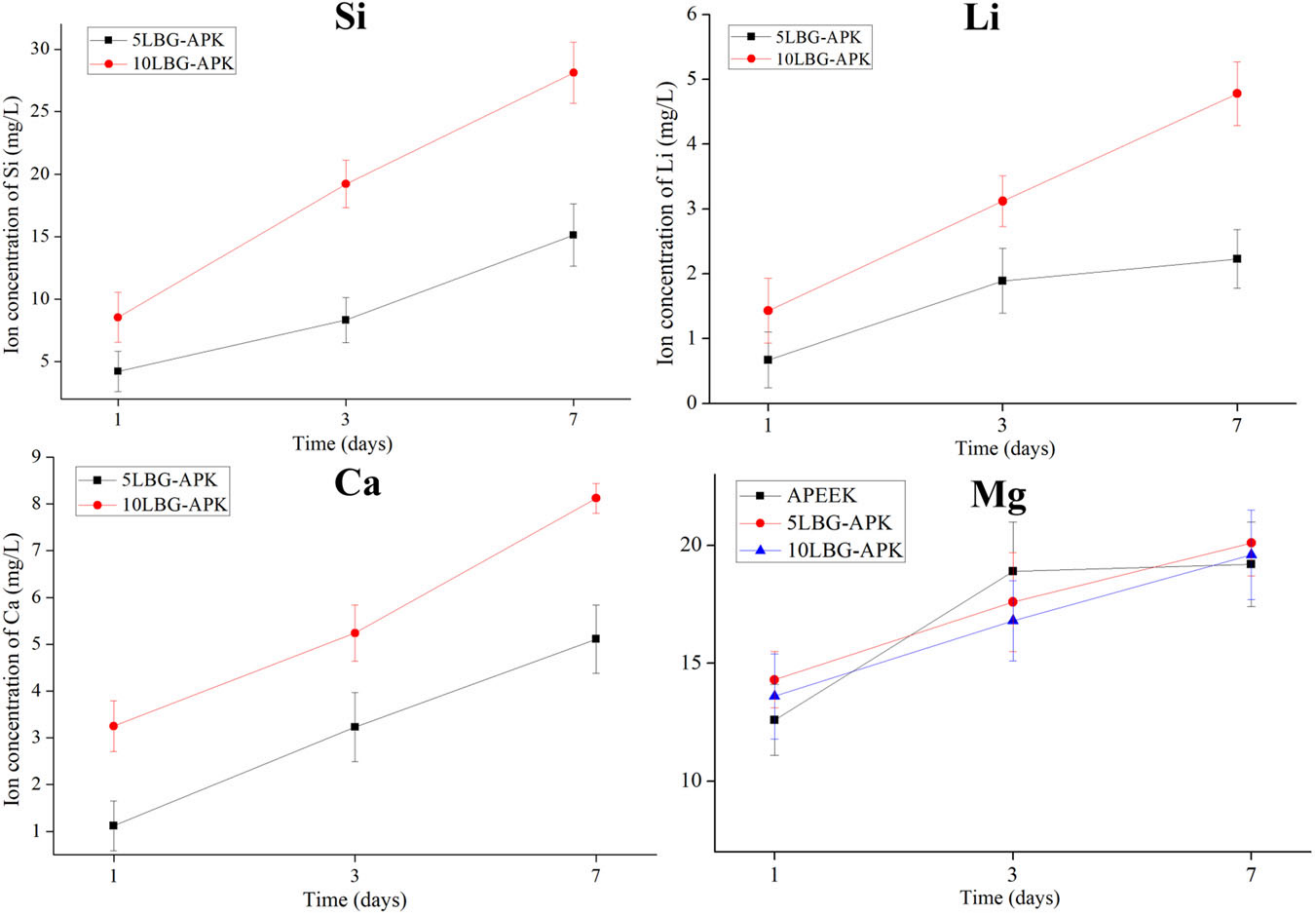
Changes of concentrations of ions (Si, Li, Ca) with time after the samples (5LBG-APK, 10LBG-APK) soaked in Tris-HCl buffer

The ion-related microenvironment of peri-implant showed important influence in the migration, adhesion, proliferation, and differentiation of BMSCs. BGNs were common ion carriers for the construction of ion-related microenvironment. After surface modification, the release of Mg, Li, Ca, and Si ions were detected after immersing in Tris-HCl buffer for 1, 3, and 7 days. (Fig. 5) 10LBG-APK showed higher Si, Ca, and Li concentration compared with 5LBG-APK in 1, 3, and 7 days. No obvious difference among APEEK, 5LBG-APK, and 10LBG-APK could be observed of Mg concentration after immersing for 1, 3, and 7 days.

**Fig. 6:**
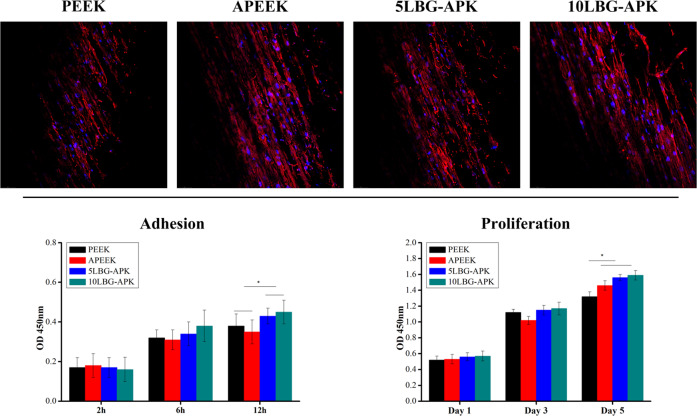
Morphology of BMSCs cultured on surface of PEEK, APEEK, 5LBG-APK, and 10LBG-APK after 24 h by CLSM. Cytocompatibility evaluation of BMSCs cultured on the surface of modified PEEK. (Adhesion evaluation for BMSCs culture on surface for 2, 6, and 12 h. Proliferation evaluation for BMSCs cultured on surfaces for 1, 3, and 5 days)

The BMSCs were cultured on the surface of PEEK, APEEK, 5LBG-APK, and 10LBG-APK. The morphologh, adhesion and proliferation of BMSCs on the surface of sample were evaluated (Fig. 6). No obvious difference could be found for the adhesion rate of BMSCs after co-culture with PEEK, APEEK, 5LBG-APK, and 10LBG-APK for 2 and 6 h. After 12 h, the adhesion rate of BMSCs co-cultured on 5LBG-APK and 10LBG-APK was higher than that of PEEK and APEEK (Fig. 6). After culture for 5 days, the cells cultured on APEEK, 5LBG-APK, and 10LBG-APK showed much higher proliferation than that of PEEK, while no noticeable difference could be observed between 10LBG-APK, 5LBG-APK, and APEEK.

**Fig. 7:**
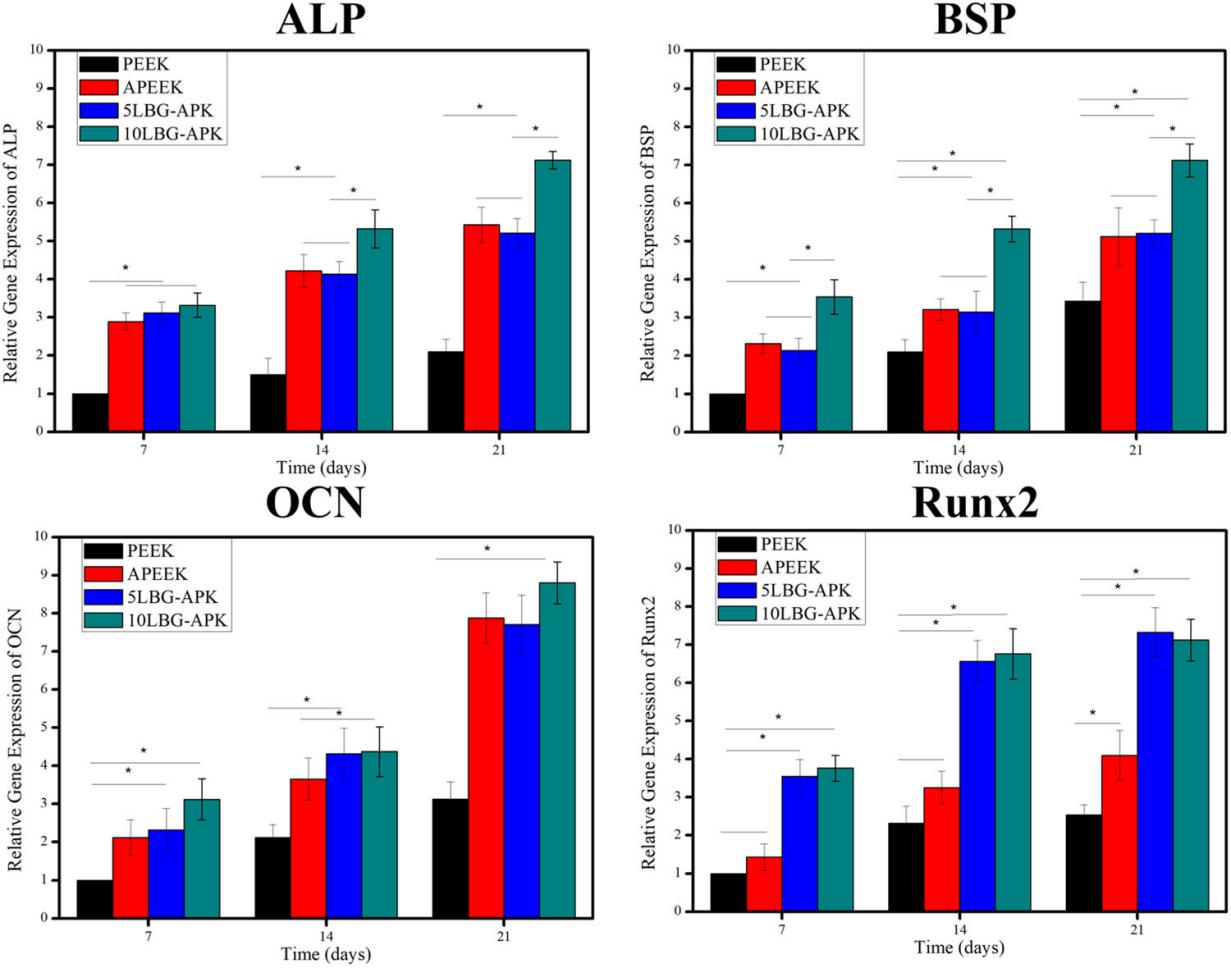
Osteogenic-related genes (ALP, BSP, Runx2, OCN) expressions of BMSCs after cultured on PEEK, APEEK, 5LBG-APK, and 10LBG-APK for 7, 14, and 21 days

The osteogenic-related gene expression of BMSCs cultured on the surface of PEEK, APEEK, 5LBG-APK, and 10LBG-APK were shown in Fig. 7. For ALP, OCN, and BSP, 10LBG-APK showed the highest expression than other samples in 7, 14, and 21 days. 5LBG-APK and APEEK showed a higher expression rate than that of pure PEEK. There is no significant difference between APEEK and 5LBG-APK. The expression of Runx2 of 10LBG-APK and 5LBG-APK was higher than that of PEEK and APEEK for 7, 14, and 21 days.

## Discussion

Nowadays, PEEK is widely accepted in hard tissue replacement and repair area due to its bone-like biomechanical (4Gpa) and biocompatible properties. However, the chemical inertness of PEEK limited the potential of direct bone bonding, osteogenesis and osteointegration property which may lead to the unsatisfactory outcomes in vitro or clinical studies [11]. To overcome this disadvantage, the BSA/Li-BGN nanocomposites was coated on the surface through dip-coating methods in our research.

Li-BGN was fabricated through a modified Stöber methods [10]. BSA hydrogel was formed by salt-induced cold gelation [12]. With the addition of magnesium chloride and heating at 55~80 °C for more than 2 h, the composite hydrogel coating showed homogeneous structure on the surface of PEEK.

The albumin coating film could support the adhesion and proliferation of the BMSCs with the degradation of the protein and the release of Mg ions. The ionic dissolution products from BG could improve the differentiation of BMSCs cultured on the surface of implants [13].

After coating with BSA and BSA/BGN layer, the roughness and hydrophilicity of the surface was increased. The improvement of the hydrophilia would be much easier for the adsorption of biomacromolecules that lead to the improvement of adhesion. An early study showed that fibronectin would be adsorbed to BSA hydrogels due to the exposure of the BSA binding sites in our acidic medium induces a change in the protein’s surface charge, which facilitates protein adsorption [7]. The adhesion rate of stem cells would be improved with the improvement of the roughness of the surface.

The ion-related microenvironment showed great influence on the cell response to the artificial implants [14]. In our study, the ion-related microenvironment was constructed with dissolution of Mg from BSA hydrogel and the ionic dissolution product from lithium containing bioglass nanospheres. There was no obvious difference in the Mg ion concentration of APEEK, 5LBG-APK, and 10LBG-APK. 10LBG-APK showed higher concentration of ionic dissolution products (Li, Ca, and Si) from bioglass nanospheres. Some of osteo-related gene (ALP, OCN, BSP) showed similar tendency, 10LBG-APK showed highest expression rate due to the higher concentration of Ca, Si, and Li ions. 5LBG-APK showed similar expression rate with APK may be because of the similar ion concentration of Mg ions from the BSA substrate. But the expression rate of Runx2 of 5LBG-APK and 10LBG-APK were significant higher than that of PEEK and APEEK. This may due to the concentration of Li ions [15]. It has been demonstrated that Li ions acts on the proliferation and differentiation of bone marrow mesenchymal stem cells, stimulating osteogenesis by activating different Wnt and Hedgehog (Hh) signaling pathways and inhibiting the enzyme glycogen synthase kinase-3β which showed important influence on the expression of Runx2.

In summary, nanocomposite coating composed of albumin and Li-BGN constructed a microenvironment enriched Mg, Ca, Li, and Si, BMSCs cultured on the composite coating showed enhanced adhesion and proliferation rate, higher osteogenesis related gene expression rate compared with pure PEEK.

## Conclusions

In this research, a coating of BSA and Li-BGN layer was constructed by dip-coating methods. The Li-BGN were mono-dispersed on the BSA substrate. The mechanical property and micro-nano structure of the surface could improve the adhesion of BMSCs. The degradation and dissolution products of BSA layer could accelerate the proliferation of BMSCs. The Mg, Li, Ca, and Si ions release from the BGNs in the BSA layer could induce the new bone formation. These results indicated that the composite surface could improve the adhesion, proliferation, and differentiation of BMSCs cultured on the surface of the modified implant.

## Data Availability

Data will be available upon request.
